# New Combined Medical Treatment With Etilefrine and Octreotide for Chylothorax After Esophagectomy: A Case Report and Review of the Literature

**DOI:** 10.1097/MD.0000000000002214

**Published:** 2015-12-11

**Authors:** Yu Ohkura, Masaki Ueno, Toshiro Iizuka, Shusuke Haruta, Tsuyoshi Tanaka, Harushi Udagawa

**Affiliations:** From the Department of Gastroenterological Surgery (YO, MU, SH, TT, HU), and Department of Gastroenterology (TI), Toranomon Hospital, Tokyo, Japan.

## Abstract

Postoperative chylothorax is a rare but well-known complication of general thoracic surgery. Medical treatment of chylothorax was reported in the past, but there is still considerable controversy on the appropriate management strategies.

Two patients with esophageal cancer underwent esophagectomy, 2-field lymph node dissection, and resection of thoracic duct together with ileocolic reconstruction via the retrosternal route at our hospital. Chylothorax developed on the 32nd postoperative day (POD) in 1 patient and the 12th POD in the other, manifesting as a change in the character of thoracic drainage to turbid white. Both were immediately started on octreotide (300 μg/ day) and etilefrine (120 mg/day). When the amount of pleural effusion decreased to <50 mL/day, we performed pleurodesis with Picibanil (OK432). Thereafter, the patients gradually made satisfactory progress and resumed oral food intake, and the thoracotomy tubes were eventually removed. They have remained recurrence-free at the time of writing.

In this report, we demonstrated the clinical efficacy of etilefrine for the management of postesophagectomy chylothorax. New medical treatment options for this condition are now broad and the usefulness of combined therapy consisting of a sclerosing agent, etilefrine, and octreotide is underscored, regardless of the status of the thoracic duct.

## INTRODUCTION

Postoperative chylothorax after esophagectomy occurs relatively infrequently in about 2 %.^1^ Few studies have addressed the best treatment regimens for chylothorax.^2^ Nutritional regimens and pharmacological and surgical therapies exist but there is still a lack of a clear consensus on the optimal management of chylothorax.^3^ Surgery is not always inevitable due to the overwhelming availability of nutritional regimens and pharmacologic therapies. The advantage of medical management alone is that the patient does not have to undergo reoperation, which can result in complications. In addition, it may also be difficult to identify the site of leakage during surgery. We investigated the usefulness of etilefrine, a sclerosing agent, alone and combined therapies consisting of etilefrine and octreotide to broaden the medical treatment options for postesophagectomy chylothorax. From our experience, combined therapy consisting of etilefrine and octreotide may be optimal for the initial treatment of chylothorax after esophagectomy. This report was approved by our hospital's institutional review board results.

## CASE PRESENTATION

### Case 1

**FIGURE 1:**
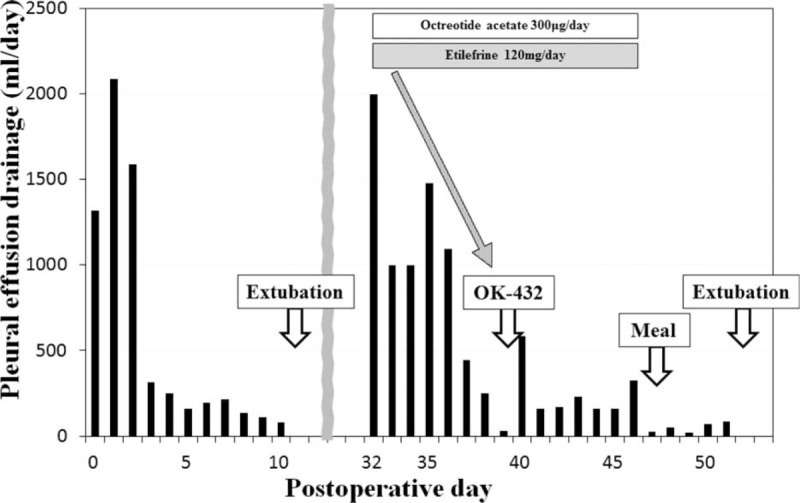
Clinical course and chyle leakage output in a 53-year-old man who underwent esophagectomy with thoracic duct resection.

The patient was a 53-year-old man who was diagnosed with early stage cancer of the upper esophagus by an upper gastrointestinal series in 2014. Upper gastrointestinal endoscopy showed a shallow depressed lesion (0–IIc), about 2 cm in diameter, for which endoscopic submucosal dissection (ESD) was performed. Pathologic findings showed squamous cell carcinoma of the esophagus, 0–IIc, 25 × 12 mm, depth T1a–MM (M3), ly0, v0, pHM1, pVN0. After ESD, surgical resection was performed because of suspected lymph node metastasis. We performed esophagectomy, 2-field lymph node dissection, and ileocolic reconstruction via the retrosternal route. The thoracic duct was inadvertently resected together with the surrounding swollen lymph nodes. Figure 1 shows the amount of pleural drainage through the thoracotomy tube postoperatively. Although >1500 mL/day of serosanguinous exudate was drained on the first few days after surgery, the amount of effusion decreased thereafter and the thoracotomy tube was removed on the 11th postoperative day (POD). The patient had an uncomplicated postoperative course and was discharged from the hospital on the 19th POD.

However, on the 32nd POD, he presented to our hospital because of dyspnea. Computed tomography (CT) scan of the chest revealed bilateral pleural effusion that was greater on the right. A thoracic tube was inserted and 2000 mL of chylous pleural effusion was drained by thoracentesis. Immediately, we started octreotide (300 μg/day) and etilefrine (120 mg/day) as medical management. The chylous pleural effusion was gradually reduced, and when the amount was <50 mL/day on the 39th POD, we performed pleurodesis with Picibanil (OK432). After that, the patient made satisfactory progress and resumed oral food intake on the 47th POD. The thoracotomy tube was removed the on 52nd POD. He remained recurrence-free after 1 year of follow-up.

### Case 2

**FIGURE 2:**
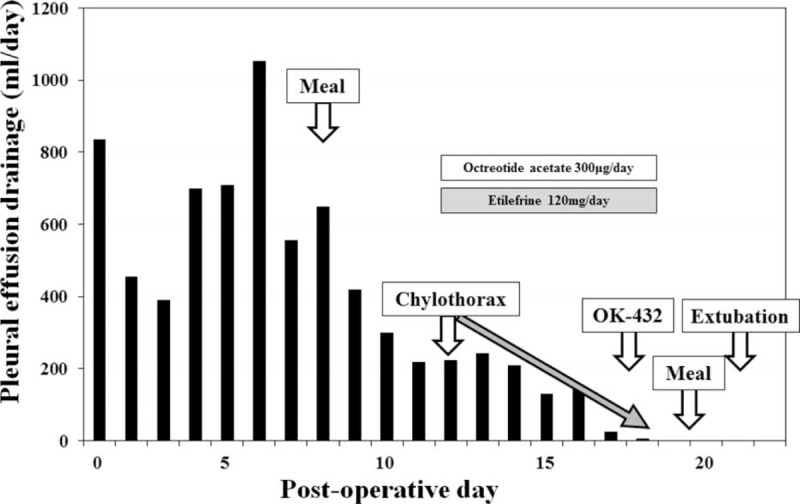
Clinical course and chyle leakage output in a 41-year-old man who underwent esophagectomy with thoracic duct resection.

The patient was a 41-year-old man who presented to our hospital in 2014 with a chief complaint of dysphagia. Upper gastrointestinal endoscopy showed advanced cancer at the esophagogastric junction that pathological diagnosed as adenocarcinoma, tub1–muc. He was given 2 cycles of neoadjuvant chemotherapy (TS-1 + CDDP), followed by esophagectomy, 2-field lymph node dissection, resection of the thoracic duct, and ileocolic reconstruction via the retrosternal route. Figure 2 shows the daily postoperative drainage through the thoracotomy tube. On the 8th POD, the amount of effusion gradually decreased and the patient started oral food intake. However, on the 12th POD, we noted a change in the character of thoracic drainage from serosanguinous to turbid white. After a diagnosis of chylothorax was made, we immediately started octreotide (300 μg/day) and etilefrine (120 mg/day) as medical management. The chylous pleural effusion was gradually reduced and when the amount was <50 mL/day on the 18th POD, we performed pleurodesis with Picibanil (OK432). Thereafter, the patient made satisfactory progress and resumed oral food intake on the 19th POD. The thoracotomy tube was removed on the 21st POD. He has remained recurrence-free after 8 months of follow-up.

## DISCUSSION

Postoperative chylothorax is a rare but well-known complication of general thoracic surgery.^4–7^ Initial treatment of chylothorax is usually conservative. The cornerstone of treatment is adequate fluid and electrolyte replacement, along with appropriate nutrition (with medium-chain triglyceride diet) and drainage of the effusion. If conservative treatments are not successful, pleurodesis is performed. Surgery has not always been the inevitable option because of the overwhelming availability of nutritional regimens and pharmacologic therapies. The advantage of medical management alone is that the patient does not have to undergo reoperation, which might result in complications. In addition, it may also be difficult to identify the site of leakage during surgery.

Recently, we have been performing simple open or thoracoscopic ligation of the thoracic duct. However, it is more difficult to completely resolve a chylothorax if the thoracic duct was resected, rather than preserved. As seen in our 2 cases in which the thoracic duct was inadvertently resected, the site of leakage has to be identified instead of merely performing simple ligation. Usually, preoperative identification of the leakage site entails doing a lymphogram, which is an uncertain and cumbersome procedure.

We investigated the value of etilefrine in order to broaden the medical treatment options for the management of postesophagectomy chylothorax. To the best of our knowledge, the efficacy of etilefrine by itself for the management of chylothorax has been reported only twice in the literature. Both reports were reported by Guillem in 1999 and 2004. Etilefrine is an α-adrenergic and β-adrenergic drug that is used in postural hypotension, priapism, etc. Through its sympathomimetic action, etilefrine induces contraction of the smooth muscles of the thoracic duct or main lymphatic duct, leading to a narrowed lumen. As a result, the amount of chyle will be reduced and the site of injury will be repaired. Etilefrine has few side effects such as headache, tachycardia, elevated blood pressure, anxiety, and flushes.

Guillem et al first reported their experience of etilefrine as medical treatment for 13 cases of chylothorax after esophagectomy from 1999 to 2015 (Table 1). Eleven patients underwent esophagectomy with preservation of the thoracic duct, whereas the remaining 2 underwent inadvertent resection of the thoracic duct. Therapy was etilefrine alone in 9 patients; octreotide plus etilefrine in 1 patient; and combination therapy consisting of octreotide, etilefrine, and a sclerosing agent (OK–432) in 3. This report showed the usefulness of a regimen consisting of etilefrine. Single-agent therapy with etilefrine or a combination of etilefrine and octreotide has been shown to reduce chyle leakage, but there was a high (40%) incidence of recurrence that eventually needed reoperation. On the other hand, all 3 patients who received a combination of etilefrine regimen and pleurodesis did not need reoperation, and recurrence was not observed. Similar to our cases, even if the pleural effusion was resolved by medical treatment with etilefrine or octreotide, pleurodesis with OK-432 was required to prevent the recurrence of chylothorax.

**TABLE 1:**
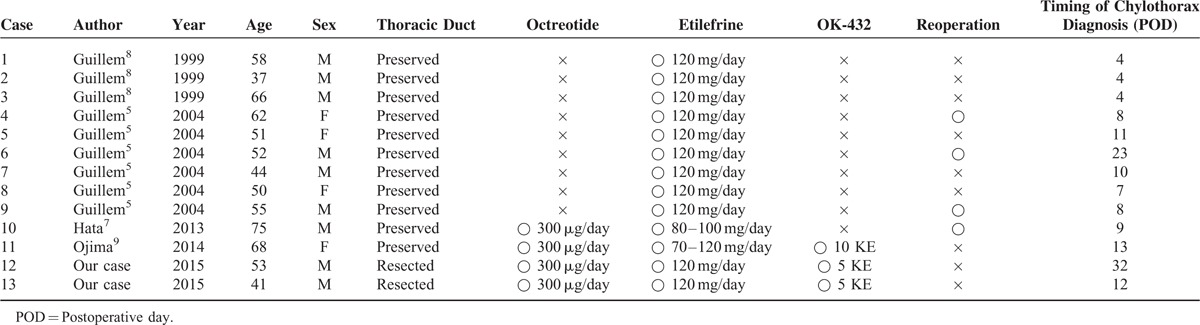
Characteristics of Patients With Postesophagectomy Chylothorax Who Were Treated With a Regimen Containing Etilefrine

In the past report, the thoracic duct was preserved in all 11 patients. Ojima et al reported that it was difficult to use combined therapy consisting of etilefrine, octreotide, and OK-432 for patients who underwent thoracic duct resection and that this therapy for chylothorax was indicated for patients with a preserved thoracic duct.^9^ However, in our 2 cases with a resected thoracic duct, this combination therapy was successful. The major limitation of our study was enrollment. This is a single-center, case study, and thus, a multicenter study examining a larger number of cases is necessary. To the best of our knowledge, this is the first report on the successful combination medical treatment for postesophagectomy chylothorax due to a resected thoracic duct. Accumulation of cases would allow more precise analysis, which might show strong effectiveness of this new combined treatment.

## CONCLUSIONS

Postesophagectomy chylothorax may be successfully managed by combined therapy consisting of etilefrine, octreotide, and OK-432, regardless of the status of the thoracic duct. Combined therapy consisting of an etilefrine regimen may be optimal for the initial treatment of chylothorax after esophagectomy.
